# Reducing intravenous thrombolysis delay in acute ischemic stroke through a quality improvement program in the emergency department

**DOI:** 10.3389/fneur.2022.931193

**Published:** 2022-09-26

**Authors:** Guangxiong Yuan, Hong Xia, Jun Xu, Chen Long, Lei Liu, Feng Huang, Jianping Zeng, Lingqing Yuan

**Affiliations:** ^1^Department of Emergency, Xiangtan Central Hospital, Xiangtan, China; ^2^Department of Cardiology, Xiangtan Central Hospital, Xiangtan, China; ^3^National Clinical Research Center for Metabolic Diseases, Department of Metabolism and Endocrinology, The Second Xiangya Hospital, Central South University, Changsha, China

**Keywords:** stroke, intravenous thrombolysis, door-to-needle time, quality improvement program, thrombolysis delay

## Abstract

**Objective:**

This study aims to investigate the effectiveness of a quality improvement program for reducing intravenous thrombolysis (IVT) delay in acute ischemic stroke (AIS).

**Materials and methods:**

We implement a quality improvement program consisting of 10 interventions for reducing IVT delay, including the establishment of an acute stroke team, standardized management of stroke teams, popularization of stroke and its treatment, emergency bypass route (BER), the achievement of computed tomography (CT) priority, no-delay CT interpretation, intravenous thrombolysis on the CT table, payment after treatment, whole recording, and incentive policy. We retrospectively analyzed the clinical time and outcome data of AIS patients treated with IVT in pre-intervention (108 patients) and post-intervention groups (598 patients), and further compared the differences between the non-emergency bypass route (NBER) and BER in the post-intervention group.

**Results:**

The thrombolysis rate increased from ~29% in the pre-intervention group to 48% in the post-intervention group. Compared with the pre-intervention group, the median of door-to-needle time (DNT) was greatly shortened from 95 to 26 min (*P* < 0.001), door-to-CT time (DCT) was noticeably decreased from 20 to 18 min (*P* < 0.001), and onset-to-needle time (OTT) significantly declined from 206 to 133 min (*P* = 0.001). Under the new mode after the intervention, we further analyzed the IVT delay difference between the NBER (518 patients) and BER groups (80 patients) from the post-intervention group. The median values of DNT (18 vs. 27 min, *P* < 0.001), DCT (10 vs. 19 min, *P* < 0.001), and OTT (99 vs. 143 min, *P* < 0.001) showed significant reductions in the BER group. The quality improvement program under the emergency platform successfully controlled the median of DNT to within 26 min.

**Conclusions:**

Collectively, the BER mode is a feasible scheme that greatly decreased DNT in AIS patients, and the secret to success was to accomplish as much as possible before the patient arrives at the emergency room.

## Introduction

Stroke is one of the most important public health problems in the world (1). In China, it is the main cause of disability and death in urban and rural areas, constituting almost one-third of the total number of deaths from stroke worldwide (2). Ischemic stroke accounts for 70% of all strokes (3). Early intravenous thrombolysis significantly improves the chance of recovery in patients with acute ischemic stroke (AIS) in a time-dependent manner (4–7). Therefore, reducing the delay of treatment has become the focus of many studies in recent years (8, 9), and associated studies have shown that shortening the door-to-needle time (DNT) can effectively reduce the delay in the hospital and improve the thrombolysis rate of stroke patients (10–12). The American Heart Association/American Stroke Association (AHA/ASA) guideline recommends DNT ≤60 min (6), but research suggests that only 27% of patients received intravenous thrombolysis within 60 min from hospital arrival (13).

At present, the relatively fastest DNT in the world is 20 min in the Helsinki model and 25 min in the Melbourne model (6, 14). Compared with the developed countries, some measures reducing the delay of thrombolysis cannot be fully adapted to all regions in the developing countries. Therefore, published data for shortening DNT have shown some gaps, variations, and inconsistencies in the results achieved by many teams (15, 16). In China, the previous electronic medical records of patients in other medical institutions cannot be obtained in advance, as they are in the Helsinki model (14). Similarly, due to the problem of medical insurance, it is also impossible to pre-mix expensive drugs, such as alteplase (rt-PA). In addition, the doctor–patient relationship in our country is in a tense situation at this time, and the risk of thrombolysis treatment is high. Therefore, every link between doctor–patient communication and decision-making is particularly important. On this basis, we have been actively exploring a kind of thrombolysis model suitable for the national conditions of China.

In this study, we analyzed the association data of DNT between pre-intervention and post-intervention modes at Xiangtan in China, and introduced the experience of our center's thrombolysis model to improve the delay time of patients' treatment from three aspects: stroke education, team training, and simplification of the thrombolysis process. We expect to provide a medical therapeutic reference to more developing countries and regions with similar environments in health care systems.

## Materials and methods

### Patient selection

In the retrospective chart research, the AIS patients who were treated with intravenous thrombolysis within 6 h of onset, between February 2016 and September 2019, at the Xiangtan City Central Hospital emergency green channel were enrolled. Urokinase treatment within 6 h of stroke onset was approved in China. Therefore, according to the Chinese guidelines and the medical environment in China, patients receive intravenous thrombolysis with rt-PA within 3 h of onset, rt-PA or urokinase within 3–4.5 h (patients decide themselves), and urokinase within 4.5–6 h. Patients were divided into pre-intervention (from February 2016 to July 2017) and post-intervention groups (from August 2017 to September 2019). In-hospital and transferred stroke patients were excluded from this study.

### Data collection

The recorded clinical data of patients were demographics (age and gender) and baseline National Institutes of Health Stroke Scale (NIHSS) scores. The thrombolytic rate was calculated as the number of patients with AIS who received thrombolytic therapy divided by the number of patients with AIS who were admitted through the emergency green channel. In addition, the stroke teams who were equipped with an accurate time recording instrument recorded the entire process of admission and thrombolysis in the patient through the stroke center thrombolytic execution schedule, which is filled on paper as soon as the stroke warning started, and then all the time points are manually entered into an Excel sheet to count the time consumption of each procedure. The time points include DNT, onset-to-needle time (OTT), onset-to-call for help time (OTC), door-to-CT time (DCT), duration in ED (emergency department), ED-to-CT time, CT-to-needle time (CNT), and the percentage of patients treated within 20, 30, and 60 min of arrival were statistically analyzed. Stroke outcomes recorded were 24-h NIHSS after thrombolysis, symptomatic intracerebral hemorrhage (sICH) ≤36 h, IVT complication, in-hospital mortality, and stroke mimics thrombolysis.

### Previous Xiangtan Central Hospital thrombolysis model (pre-intervention)

**Figure 1:**
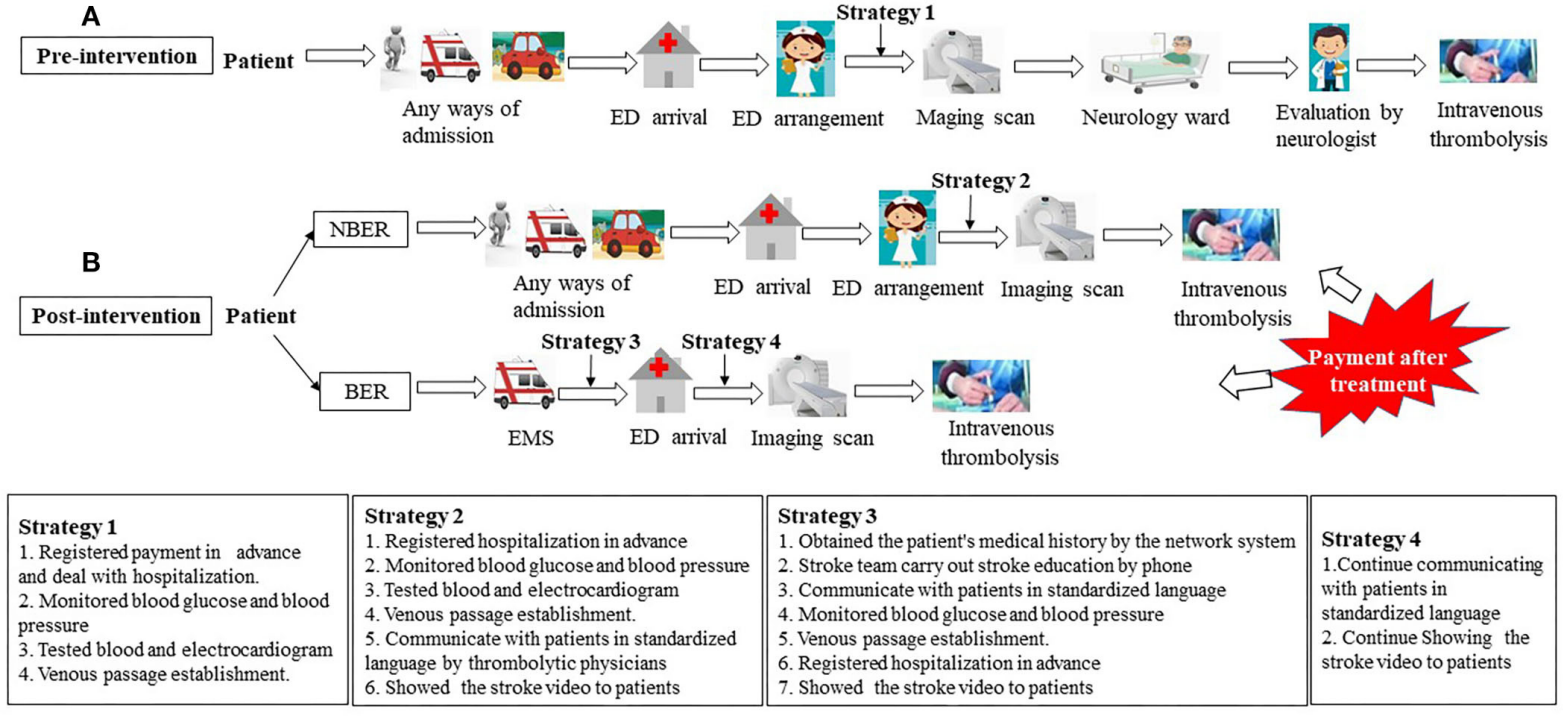
Pre-intervention and post-intervention processes for intravenous thrombolysis in AIS patients. **(A)** Pre-intervention processes for intravenous thrombolysis in AIS patients; **(B)** Post-intervention processes for intravenous thrombolysis in AIS patients. ED, emergency department; NBER, non-emergency bypass route; BER, emergency bypass route; EMS, emergency medical service.

The operation process of the previous Xiangtan Central Hospital thrombolysis model is shown briefly in Figure 1A. Before August 2017, the paramedics evaluated AIS patients first. Upon arrival at our emergency department, patients were served to the emergency green channel of stroke, and further evaluated by emergency physicians. The following measures were completed in the emergency department: (1) registered payment in advance and deal with hospitalization; (2) monitored blood glucose and blood pressure; (3) tested blood and electrocardiogram; and (4) establish a venous passage. An urgent reservation for computed tomography (CT) scan of the brain was performed if the patient was considered to be potentially eligible for acute revascularization treatment. A stroke neurologist was alerted simultaneously. After the CT was finished, the AIS patient was transferred to the neurology high-dependency ward. A neurologist reviewed the patient and CT scan, performed the NIHSS scores, and subsequently talked to the patient's family and asked the family to sign for intravenous thrombolysis. Finally, intravenous thrombolysis was performed in the neurology ward.

### Post-intervention model

In order to reduce the delay of thrombolysis in AIS patients, we implemented a quality improvement program consisting of 10 interventions for reducing treatment delay (Table 1). In detail, the stroke team was reorganized and led by the emergency department, replacing the neurology department (17). Because the emergency platform is generally the only 24-h continuous operation platform in the hospital, the emergency platform can not only effectively integrate the resources of the whole hospital medical staff (the departments of neurology, nursing, radiology, laboratory, interventional, neurosurgery, and rehabilitation medicine), but can also efficiently operate the whole stroke emergency aid system. One of the various effective intervention measures was to educate the medical staff about stroke (18). Therefore, EMS, thrombolytic physicians, and nurses were systematically educated to quickly identify stroke patients, master the standardized process for thrombolysis, and define the responsibilities and roles of every member of the participating departments. Also, physicians were required to use standardized language to communicate with the patient's family at each moment during the process.

**Table 1:** Systemic interventions incorporated into the standard operating procedures.

**Intervention steps**	**Description**
Establishment of acute stroke team	1) A team led by the emergency department, integrating medical staff from the departments of neurology, nursing, radiology, laboratory, interventional, neurosurgery and rehabilitation medicine
	2) Set up a network working group among stroke team members
Standardized management of stroke teams	1) Working hours are 24 h a day, 7 days a week
	2) Set up a special stroke telephone number
	3) Establishment of an electronic thrombolytic database
	4) Use standardized language to communicate with the patient's family (Supplementary Table S1)
	5) Train thrombolytic physicians and nurses to quickly identify stroke patients, and master the standardized process for thrombolysis. The stroke team medical staff can only be on duty if they meet the requirements through an assessment
	6) Conduct monthly quality control meeting to optimize the thrombolysis process
Popularizing stroke and its treatment	1) Made education stroke picture and vide (Supplementary material V1)
	2) Educated dispatchers and EMS personnel to quickly identify and high-priority dispatch stroke patients
	3) Carried out stroke education twice a month in the community
	4) Established an official WeChat account to publicize stroke knowledge
	5) Encouraged primary hospitals to carry out intravenous thrombolysis therapy and join in the dissemination of stroke information
Emergency “zero pause”—emergency bypass route (BER)	1) In an ambulance, establish an intravenous cannula, draw blood, monitor blood glucose and blood pressure levels, complete an electrocardiogram examination by EMS
	2) Stroke team carries out stroke education and communicate with the patient's family members by phone and fills out the thrombolysis implementation table
	3) Uploaded the patients' medical records to the network for the working group, showed the stroke education video to the patient's family members during transportation to the emergency department
The achievement of CT priority	The patient entered the green channel, and CT priority was achieved for AIS patients eligible for intravenous thrombolysis
No-delay CT interpretation	The physician specializing in strokes interprets the CT scan, and does not wait for formal radiology report
Intravenous thrombolysis on the CT table	After a CT scan of the head is finished, a tPA or urokinase bolus (intravenous thrombolysis with tPA within 4.5 h of onset, and urokinase within 6 h) was subsequently administered in the CT room. After starting the continuous infusion, patients were directly transferred to the emergency stroke ward in our hospital
Payment after treatment	After the patient arrives in the hospital, it is not necessary to pay the treatment fee immediately. The patient's family only needs to sign the consent for the corresponding treatment
Whole recording	Record the entire diagnosis and treatment process and accompanied by stroke physicians and nurses in the whole process. The special stroke quality control team listens to the recorded data to check whether the AIS patient was quickly identified and if the staff communicated with the patients using the standardized language
Incentive policy	When the stroke team has reduced the DNT to less than 60 minutes, the corresponding small bonus will be awarded

We implemented 24-h work schedules and set up a special stroke telephone number, which was widely distributed to the EMS units to obtain directly the history of suspicious stroke patients. The thrombolysis physician of the stroke team requests communication with the primary informants at the scene through a mobile phone, and records the time point associated with the onset of the disease through the paper stroke center thrombolytic execution schedule (19). Subsequently, the stroke team is alerted about an incoming thrombolysis candidate. Upon arrival, the suspected stroke patients are immediately entered into the emergency stroke green channel, and the thrombolytic physician performs rapid physical examination and neurologic evaluation using the NIHSS for preliminary diagnosis. Meanwhile, the CT priority is assigned to patients eligible for intravenous thrombolysis, and the thrombolytic physician shows a stroke education video to patients and explains the possibility of thrombolytic therapy for patients on the way to the CT room. The physician specializing in strokes interprets the CT scan and does not wait for the formal radiology report. Subsequently, rt-PA (20) or urokinase (21) is administered in the CT room. Besides, the AIS patient is accompanied by stroke physicians and nurses in the whole door-to-needle process. The stroke team records the entire diagnosis and treatment process to check whether the AIS patient was quickly identified and if the staff communicated with the patients using standardized language. The special stroke quality control team conducts monthly quality control meetings to optimize the thrombolysis process. After the patient arrives at the hospital, it is not necessary to pay the treatment fee immediately. The patient's family only needs to sign the consent for the corresponding treatment. We also motivate the stroke team to reduce the DNT to <60 min with a little reward.

It is worth mentioning that there are still many AIS patients who do not receive intravenous thrombolysis within 6 h. This is due to the ignorance of stroke symptoms. Thus, we took the following measures to make patients understand stroke symptoms quicker. First, our stroke team members carry out stroke education activities in the community two times a month. They distribute paper materials about stroke education to popularize knowledge about stroke, and train the community residents to master the key points of stroke first aid. Second, the stroke team established a WeChat public account for stroke education and regularly update the content to publicize knowledge related to stroke. Third, the team made videos related to stroke education and put them on internet-related platforms to publicize the development of stroke disease and treatment time dependence. At the same time, for the patients admitted to our hospital for stroke treatment, we develop a more detailed stroke education and follow-up plan, and suggest that they inform their relatives and friends regarding their medical experience and knowledge of stroke. In addition, we conduct medical consortium activities that promote primary medical units to carry out stroke intravenous thrombolysis and regular community stroke education work. In this way, residents in remote areas can also learn more about stroke and seek help in a timely manner.

In addition, we developed standard operating procedures for all patients eligible for intravenous thrombolysis to reduce intravenous thrombolysis treatment delays. The two procedures [emergency “zero” pause-emergency bypass route (BER) and non-emergency bypass route (NBER)] are shown in Figure 1B. NBER means that the patient enters the green channel, and if a suspected AIS patient meets any of the FAST (Face-Arm-Speech-Time) items when arriving at the emergency department, a rapid physical examination is performed immediately, and the patient is assessed for NIHSS score by a thrombolytic physician of the stroke team. The nurse of the stroke team performs a rapid monitoring of blood glucose and blood pressure, draws blood, conducts an electrocardiogram examination, and establishes venous access in the emergency department. At the same time, the thrombolytic physician fills out the stroke center thrombolytic execution schedule, shows the stroke education video to the patients, and explains the possibility of thrombolytic therapy for patients on the way to the CT room. After completing a CT scan of the head, the thrombolytic physician and imaging physician browse the image without cerebral hemorrhage or new low-density foci, re-inform the family of the need for intravenous thrombolysis, obtain the signed informed consent from patient, and subsequently administer the rt-PA or urokinase in the CT room. After starting the continuous infusion, patients are directly transferred to the emergency stroke ward of our hospital.

Compared with the NBER mode, the patients in BER mode are admitted by ambulance from the EMS team. These patients are initially assessed for the NIHSS score by an EMS team member at the scene of the patient's disease onset. The EMS team is immediately replaced by a stroke team for NIHSS score correction upon arrival at the hospital. In addition, the stroke team would pick up information about the history of past and present illnesses of a suspected AIS patient on the ambulance by telephone (22), establish venous channels, draw blood, monitor blood glucose and blood pressure, complete an electrocardiogram examination by EMS on the ambulance. After arriving at the hospital, the blood drawn is sent to the laboratory department for examination through the green channel, and the patient is immediately transferred for an image scan.

### Statistical analysis

All data were analyzed by IBM SPSS 22 (IBM Corp, Armonk, NY). Some data were presented as the mean values ± standard deviations (SD). The data of non-normal distributed outcomes are shown as median values with an interquartile range (IQR). Mann–Whitney *U* or Kruskal–Wallis tests were used for statistical analysis of data. Dichotomous data were described as numbers and percentages. Differences in measures with a dichotomous outcome were analyzed with *x*^2^ tests. In all calculations, differences in values were considered statistically significant if *P* < 0.05.

## Results

### General characteristics of AIS patients in pre-intervention and post-intervention groups

**Figure 2:**
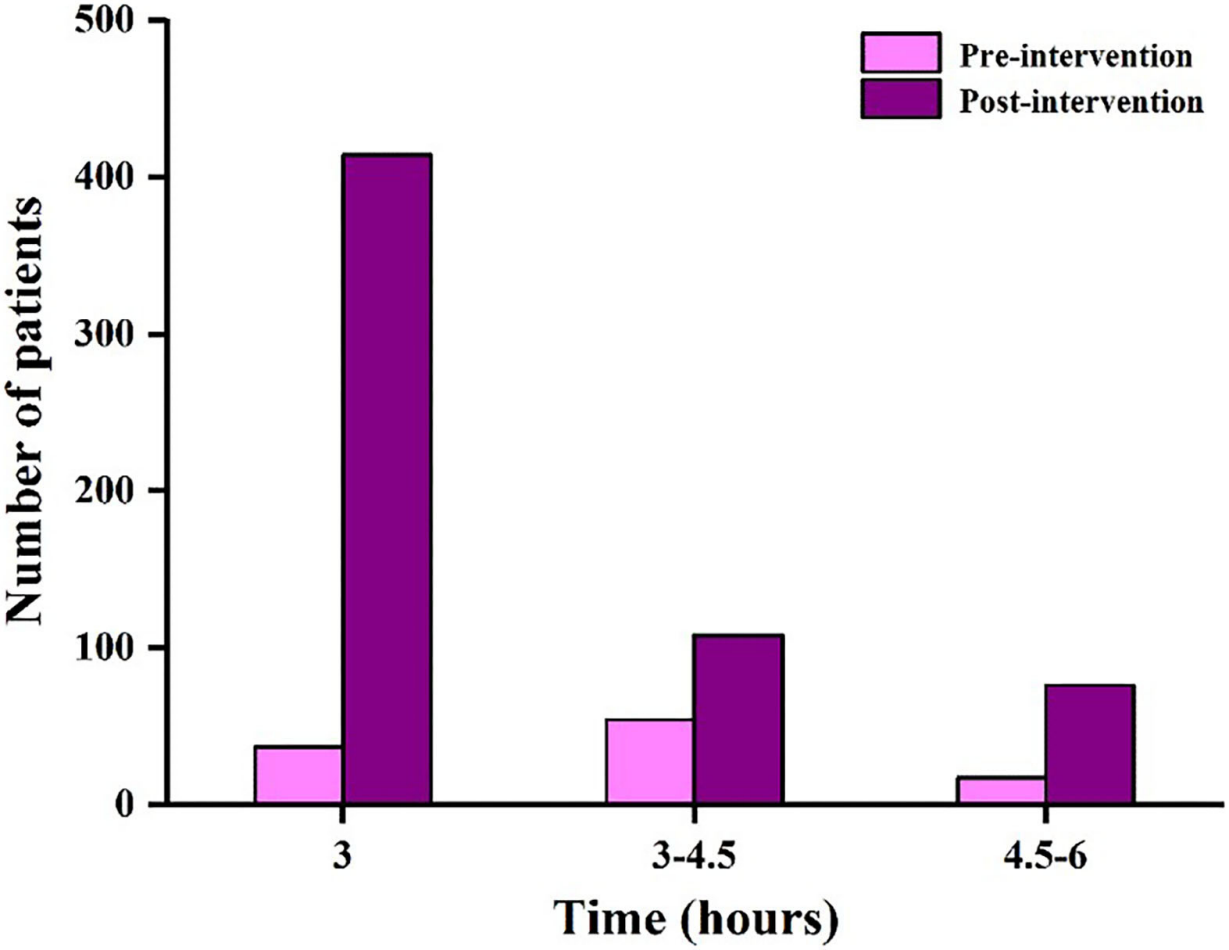
The number of AIS patients with intravenous thrombolysis in the pre-intervention and post-intervention groups at different time periods (3, 3–4.5, and 4.5–6 h).

Table 2 shows the demographics and clinical characteristics of 706 AIS patients with thrombolytic therapy in pre-intervention and post-intervention groups. From February 2016 to July 2017, a total of 108 patients received intravenous thrombolysis in the pre-intervention group. An additional 598 patients were treated from August 2017 to September 2019 in the post-intervention group. There were no significant differences in patient characteristics for age (*P* = 0.063), gender (*P* = 0.393), and baseline NIHSS scores (*P* = 0.152), which suggested that there was no variation in the included AIS patients. The thrombolysis rate of ischemic stroke patients admitted to our hospital increased from ~29 (108 cases/379 stroke alerts) to 48% (598 cases/1,258 stroke alerts). The number of AIS patients with intravenous thrombolysis in pre-intervention and post-intervention groups at different time periods (3, 3–4.5, and 4.5–6 h) are exhibited in Figure 2. In the pre-intervention group, the number of cases receiving intravenous thrombolysis within 3, 3–4.5, and 4.5–6 h was 37 cases (34.3%), 54 cases (50%), and 17 cases (15.7%), respectively. In the post-intervention group, the number of cases was 414 cases (69.2%), 108 cases (18.1%), and 76 cases (12.7%), respectively. Thus, these results indicated that more patients received intravenous thrombolysis within 3 h (34.3 vs. 69.2%, *P* < 0.001) after the interventions.

**Table 2:** Baseline characteristics, time intervals, outcome and safety measurements of patients in pre-intervention, post-intervention, NBER and BER groups.

	**Pre-intervention**	**Post-intervention**	**^a^*P*-value**	**Post-intervention**		**^b^*P*-value**
	**n = 108**	**n = 598**		**NBER (n = 518)**	**BER (n = 80)**	
Age (years)	67 (60–74)	69 (60–77)	0.063	68 (57–76)	75 (68–81)	0.009
**Gender**			0.393			0.539
Female, n (%)	38 (35.2)	238 (39.8)		212 (40.9)	29 (36.3)	
Man, n (%)	70 (64.8)	360 (60.2)		306 (59.1)	51 (63.7)	
Baseline NIHSS scores	6 (3–11)	7 (3–15)	=0.152	6 (3–14)	13 (4–18)	=0.001
Onset-to-needle time (OTT, minutes)	206 (170–250)	133 (85–206)	=0.001	143 (91–208)	99 (67–184)	< 0.001
Onset-to-call for help time (OTC, minutes)	54 (30–100)	33 (17–78)	< 0.001	40 (16–72)	23 (14–88)	=0.630
Door-to-needle time (DNT, minutes)	95 (80–117)	26 (22–32)	< 0.001	27 (23–33)	18 (13–23)	< 0.001
DNT ≤ 60 minutes, n (%)	10 (9.3)	581 (97.2)	< 0.001	501 (96.7)	80 (100)	=0.1
DNT ≤ 30 minutes, n (%)	0	419 (70.1)	< 0.001	346 (66.8)	73 (91.3)	< 0.001
DNT ≤ 20 minutes, n (%)	0	114 (19.1)	< 0.001	62 (12)	52 (65)	< 0.001
Door-to-CT time (DCT, minutes)	20 (16–24)	18 (15–22)	< 0.001	19 (16–23)	10 (9–12)	< 0.001
Duration in ED (minutes)	11 (8–13)	9 (6–12)	< 0.001	10 (7–12)	0	< 0.001
ED-to-CT time (minutes)	10 (8–12)	9 (8–11)	< 0.001	9 (8–11)	10 (9–12)	=0.004
CT-to-needle time (CNT, minutes)	75 (58–95)	7 (4–11)	< 0.001	7 (4–11)	7 (5–11)	=0.865
24 h NIHSS after thrombolysis	3 (0–9)	3 (1–11)	0.238	2 (1–9)	5 (1–15)	=0.008
Symptomatic ICH ≤ 36 h, n (%)	7 (6.5)	23 (3.8)	=0.211	20 (3.9)	3 (3.8)	=0.962
IVT complication, n (%)	20 (18.5)	93 (15.6)	=0.439	85 (16.4)	8 (10)	=0.141
In-hospital mortality, n (%)	6 (5.6)	20 (3.3)	=0.261	16 (3.1)	4 (5)	=0.582
Stroke mimics thrombolysis, n (%)	3 (2.8)	9 (1.5)	=0.591	8 (1.5)	1 (1.3)	=0.840

AIS, acute ischemic stroke; NIHSS, national institutes of health stroke scale; ED, emergency department; ICH, intracerebral hemorrhage. ^a^P-value represents compared with the pre-intervention group; ^b^P-value represents compared with the NBER group.

### Time points of AIS patients with intravenous thrombolysis in pre-intervention and post-intervention groups

**Figure 3:**
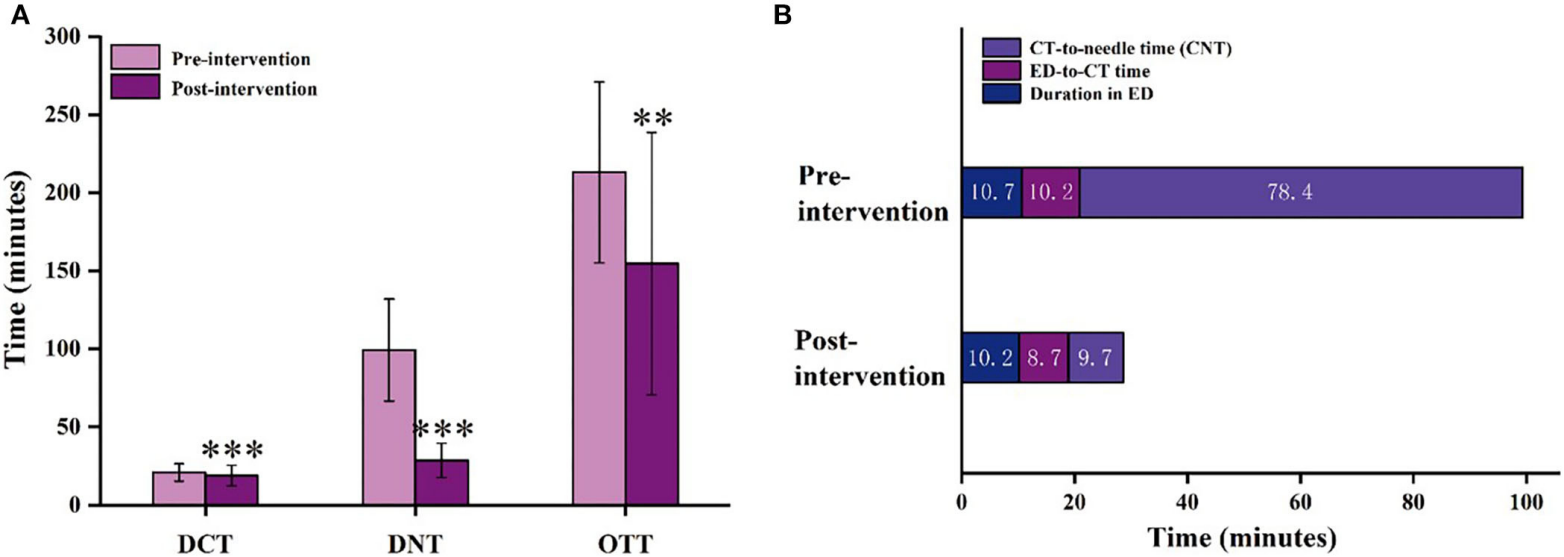
The time intervals for AIS patients with intravenous thrombolysis in the pre-intervention and post-intervention groups. **(A)** DCT, DNT, and OTT of AIS patients with intravenous thrombolysis; **(B)** Three parts of the DNT (a. Duration in ED; b. ED-to-CT time; c. CT-to-needle time [CNT].) in the pre-intervention and post-intervention groups. DCT, door-to-CT time; DNT, door-to-needle time; OTT, onset-to-treatment time; CT, computed tomography; ED, emergency department. The data are expressed as the mean ± SD. ^**^p < 0.01, ^***^p < 0.001 vs. the pre-intervention group.

The DCT, DNT, and OTT of AIS patients with intravenous thrombolysis between the pre-intervention and the post-intervention groups are shown in Figure 3A. The median (IQR) of DNT decreased significantly from 95 (80–117) min in the pre-intervention groups to 26 (22–31) min in the post-intervention groups (*P* < 0.001). In the pre-intervention groups, only 10 cases (9.3%) received intravenous treatment within 60 min. However, in the post-intervention groups, 581 cases (97.2%) received intravenous treatment within 60 min, 419 cases (70.1%) within 30 min, and 114 cases (19.1%) within 20 min (Table 2). As we all know, many complex factors affect DNT. DNT is composed of three main sections: duration in ED, ED-to-CT time, and CT-to-needle time (CNT). Figure 3B shows the duration in ED (10.7 ± 3.3 vs. 10.2 ± 3.5 min, *P* < 0.001), ED-to-CT time (10.2 ± 3.5 vs. 8.7 ± 5.5 min, *P* < 0.001), and CNT (78.4 ± 33.2 vs. 9.7 ± 8.8 min, *P* < 0.001) compared with the pre-intervention group.

The median (IQR) of DCT was significantly different between pre-intervention and post-intervention groups (Figure 3A, *P* < 0.001). Compared with the pre-intervention group, the median of DCT decreased from 20 (16–24 min) to 18 min (15–22 min). Although the reduction time was only 2 min, it is also one of the successful signs of reducing in-hospital delays. In addition, compared with the pre-intervention period, the overall delay of OTT significantly decreased from 206 to 133 min after post-intervention (Figure 3A, *P* = 0.001), and the median of OTC decreased from 54 to 33 min after post-intervention (*P* < 0.001, Table 2).

**Figure 4:**
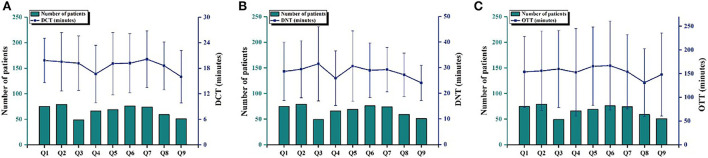
The changes in time points and the number of AIS patients with intravenous thrombolysis in the pre-intervention and post-intervention groups. Number of patients and mean DCT **(A)**, DNT **(B)**, and OTT **(C)** with standard deviation for AIS patients with intravenous thrombolysis in the post-intervention group during nine quarters (from August 2017 to September 2019, in which 3 months was a quarter, except for the ninth quarter which only had 2 months). DCT, door-to-CT time; DNT, door-to-need time; OTT, onset-to-treatment time; CT, computed tomography.

In order to better view the change in treatment delay time and the number of patients after the improvement measures, we further analyzed the number of patients and the DCT, DNT, and OTT values of AIS patients with thrombolysis in nine quarters in the post-intervention group (Figure 4). There were only 108 cases of intravenous thrombolysis in one and a half years before the improvement of the measures. However, the number of patients with intravenous thrombolysis was almost 50 cases in every quarter after the intervention. This indicated that the treatment delay in AIS patients was effectively improved, and the awareness regarding patients' early choice of medical services was gradually enhanced. The DCT, DNT, and OTT values of AIS patients in each quarter were basically in a stable state after the intervention. There was no statistical difference among quarters, which indicated that the operation of the intervention measures was feasible and mature step by step. Meanwhile, the median of 24-h NIHSS scores after thrombolysis was lower than before thrombolysis. It is worth noting that from May to July of each year [May 2018 to July 2018 (Q4) and May 2019 to July 2019 (Q8)], the DCT, DNT, and OTT values were at the lowest levels during the whole observation period. In addition, the DNT of Q4 dropped to 24 min. We speculate that the reason for this is that the patients wore fewer clothes during this period, which is beneficial to the physical examination, blood drawing, and the establishment of venous channels.

All of the above data indicated that treatment delays significantly decreased after the implementation of the intervention strategy.

### Stroke outcomes of AIS patients with intravenous thrombolysis in pre-intervention and post-intervention groups

To determine if the improvement in treatment delay can make the patient safer, the outcomes between the pre-intervention and post-intervention groups are compared. As shown in Table 2, there are no significant differences in 24-h NIHSS scores after thrombolysis (*P* = 0.238), sICH ≤ 36 h (*P* = 0.211), IVT complication (*P* = 0.439), and in-hospital mortality (*P* = 0.261). In the pre-intervention group, we treated three patients (2.8%) who turned out to have stroke mimics (one case with non-organic functional symptoms, one case with migraine, and one case with epileptic seizure). While stroke mimic thrombolysis occurred in nine (1.5%) patients after intervention (one patient with Todd's paralysis, three patients with non-organic functional symptoms, two patients with an epileptic seizure, one patient with aortic dissection, and two patients with conscious disturbance). Together, these data indicated that stroke outcomes of AIS patients with intravenous thrombolysis showed no differences after the intervention.

### AIS patients with intravenous thrombolysis in NBER and BER groups

**Figure 5:**
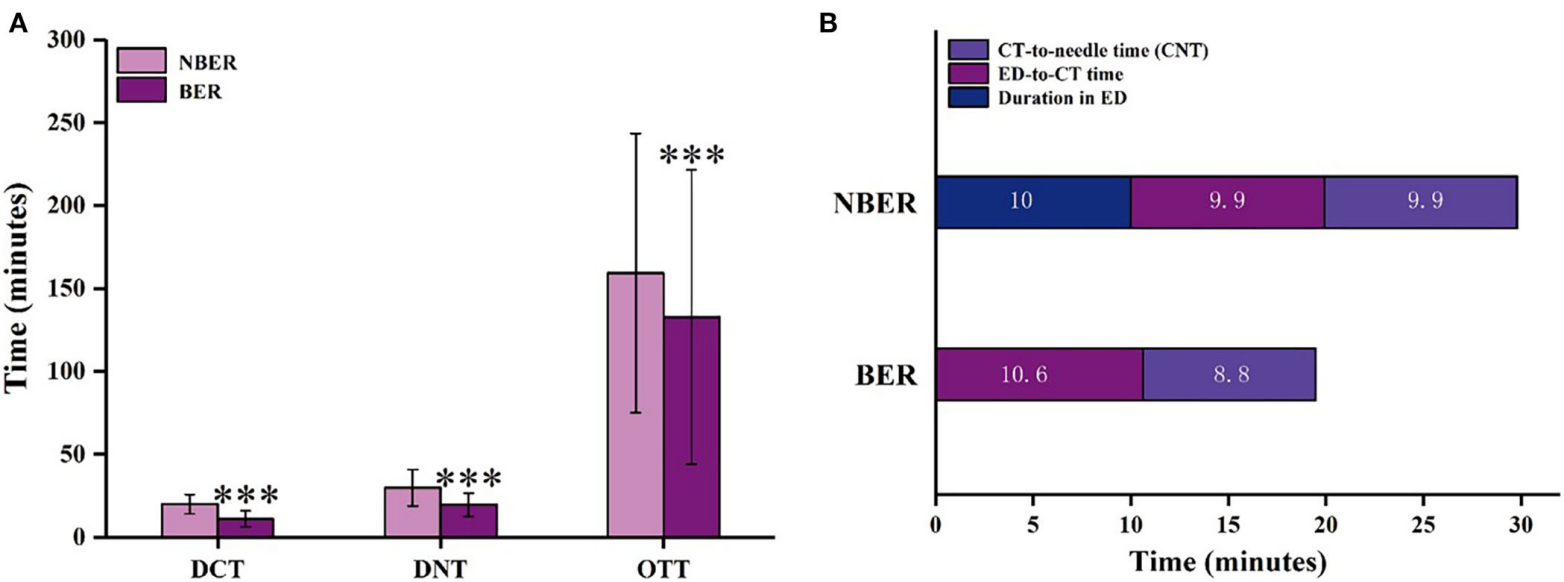
The time intervals of AIS patients with intravenous thrombolysis in the NBER and BER groups. **(A)** DCT, DNT, and OTT of AIS patients with intravenous thrombolysis; **(B)** Three parts of DNT (a. Duration in ED; b. ED-to-CT time; c. CNT) in the NBER and BER groups. NBER, non-emergency bypass route; BER, emergency bypass route; DCT, door-to-CT time; DNT, door-to-needle time; OTT, onset-to-treatment time; CT, computed tomography; ED, emergency department. The data are expressed as the mean ± SD. ^***^p < 0.001 vs. the NBER group.

Under the new mode after the intervention, the patients were divided into two groups (Figure 1B). Therefore, we further analyzed the differences in the treatment delays of intravenous thrombolysis between NBER (518 patients) and BER groups (80 patients) from August 2017 to September 2019. The demographics and clinical characteristics of the AIS patients with thrombolytic therapy for NBER and BER groups are presented in Table 1. As shown in Figure 5A, the median of DNT decreased from 27 min (IQR, 23–33 min) in the NBER group to 18 min (IQR, 13–23 min) in the BER group (*P* < 0.001). In addition, as shown in Figure 5B, there were also significant reductions in the duration of ED (10 ± 4.7 vs. 0 min, *P* < 0.001) and ED-to-CT time (9.9 ± 3.8 vs. 10.6 ± 3.2 min, *P* = 0.004). However, there was no significant difference in CNT (9.9 ± 9.2 vs. 8.8 ± 6.0 min, *P* = 0.865).

The median (IQR) of DCT declined significantly from 19 min (16–23 min) in the NBER group to 10 min (9–12 min) in the BER group (Figure 5A, *P* < 0.001), which indicates that the use of BER could reduce in-hospital delays by 9 min. In addition, the median (IQR) of OTT markedly reduced from 143 min (91–208 min) in the NBER group to 99 min (67–184 min) in the BER group (Figure 5A, *P* < 0.001), and there was no significant difference in OTC between the NBER and BER groups (40 vs. 23 min, *P* = 0.630, Table 2).

As shown in Table 2, there were eight cases of stroke mimic thrombolysis in the NBER group and one case of stroke mimic thrombolysis in the BER group. In addition, there was no significant difference in the outcome (except for 24-h NIHSS scores after thrombolysis) between the NBER and BER groups.

These results clearly established that the treatment delays for intravenous thrombolysis were decreased in the AIS patients of the BER group and that the implementation of the BER mode was safe.

## Discussion

After a quality improvement in ED, the median of DNT at Xiangtan Central Hospital was greatly shortened by 70 min, from 96 to 26 min. Our results also showed that when the reduction of DNT was achieved, more ischemic stroke patients could be treated with thrombolytic therapy. From the further analysis of monthly thrombolysis (Supplementary Figure S1), we found that the majority of patients with intravenous thrombolysis received treatment within 3 h, indicating that the intervention measures in our center significantly improved the patients' awareness of medical treatment and reduced patient treatment delay.

Currently, DNT in our center can be controlled within 30 min, and the shortest DNT is 2 min (head CT was completed at the other hospital). The Helsinki model in Finland indicated that 94% of patients receive intravenous rt-PA treatment within 60 min of hospital admission (12), while 97.2% of patients in our center receive intravenous rt-PA treatment. It also proves that emergency department cooperation, stroke education, standardized management of the stroke team, and a hospital process optimization strategy can effectively reduce the delay of treatment and improve patient outcomes (23, 24). Furthermore, our stroke center has improved DNT by reprogramming the existing programs without any additional investment, and one of the more novel highlights of our new model is that the patients' families do not need to pay the treatment fee immediately, but sign the corresponding treatment form.

When AIS patients arrive at the hospital, advance preparation by the stroke team may be beneficial (25). Once informed, the stroke team communicates with their families by phone in advance to gain their trust, and even prepares rt-PA drugs in advance to wait for AIS patients who have completed head CT. It has been reported that the most effective ways to reduce DNT are to rapidly triage and inform stroke teams, have a single-call activation system, and availability of rt-PA drugs in the emergency department (26). Our trained medical staff of the emergency department identifies possible ischemic stroke patients (18), so as to inform the thrombolytic physician at once and store rt-PA or urokinase in the emergency room thrombolytic medicine box. However, some other stroke centers store rt-PA or urokinase drugs in pharmacies or wards, which results in a delay in emergency medication for AIS patients (3). Additionally, some stroke centers wait for the results of blood tests, which leads to further extension of DNT. According to the statistics of our center, the mean time of presentation results for blood routine, coagulation routine, and liver and kidney function are 37.6, 67.6, and 71.6 min in patients with a prior channel, respectively. In our center, IVT was performed on the patients without waiting for the above test results (unless the patient has an anticoagulant or bleeding tendency and/or has a history of liver disease). After more than 2 years of observation, this strategy is recognized as safe and feasible for AIS patients with regard to the reduction of DNT, while the AHA/ASA guidelines also propose related recommendations (6, 27).

A report showed that computerized inputting of doctor's orders into the setting system reduced DCT from 34 to 19 min and even declined hospital treatment delays (28). Actually, computer set medical instructions and other instructions have been popularized in most regions. In addition, some hospitals greatly shorten the DCT by moving CT to the emergency room (ER) (8). Our center controls the median time of DCT to within 18 min. This saves 2 min compared with the pre-intervention group, which indicates that the reason for the reduction in DCT may be closely related to the duration in ED. In addition, the delay time of the pre-intervention model is mainly due to CNT. Compared with the control group, the median time of CNT was shortened by 68.7 min. Our analysis found that the reason for such a long time in the pre-intervention model is that it takes a lot of time to communicate with the patients and their families to gain their trust, make them understand the high risk of thrombolysis, and return to the ward for a reassessment of the thrombolysis treatment. However, in the post-intervention model, the stroke video was repeatedly shown to patients and their families before completing CT, so that they could quickly understand and make decisions about this disease and treatment plan. In addition, as shown in previous studies, intravenous thrombolysis is performed directly in the CT room (11, 12, 14, 29), which greatly reduces the delay time for treatment. In addition, another feature was different from the previous model, that is, all medical protocols were handled by a special stroke team, which suggested direct and effective communication with the personnel on-site.

Previous studies have shown that a special thrombolytic nurse followed by the patient's thrombolytic operation is beneficial for reducing delays (30). In the model after our intervention, a special nurse and special thrombolysis doctor accompany the patient for guidance and treatment throughout the process. Moreover, a 24-h work system in our center avoids delays in treatment due to non-working hours (15). Other strategies for shortening DNT are the whole recordings (record the entire diagnosis and treatment process, and listen to the recording data by the special stroke quality control team) and thrombolysis control meeting every month. In the meeting, we summarize the cases of DNT that took over 30 min the previous month, analyze the causes of delays in the hospital, and develop a continuous improvement protocol. It is worth mentioning that, like the Stroke Center, we also regularly train the emergency thrombolysis team (10). These measures and our united thrombolytic team have established a foundation for shortening DNT.

**Figure 6:**
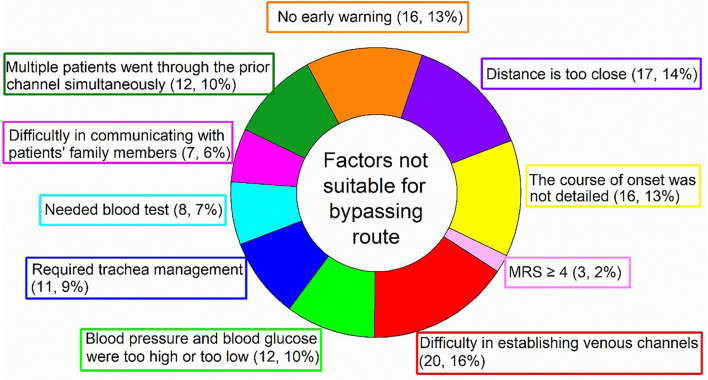
Factors unsuitable for bypassing emergency route. MRS, modified rankin scale.

In addition, the median of DNT from the AHA/ASA Get with the Guidelines–Stroke program and the Safe Implementation of Treatment in Stroke–International Stroke Thrombolysis Registry were 75 and 65 min (11), respectively, compared with 26 min in our study. To reduce in-hospital delay for AIS patients, the AHA/ASA began a national initiative in 2010 to assist hospitals to increase the proportion of patients treated with IVT within 60 min after hospital arrival. The goal of this initiative, called “Target: Stroke”, is to achieve a DNT of fewer than 60 min in at least 50% of acute ischemic stroke patients (12, 13, 31). Bypassing emergency route further reduces hospital delay and DNT, and leads to faster intravenous thrombolysis treatment for AIS patients (7). Pre-notification of stroke by emergency medical services has been endorsed by national recommendations and implemented in some developed countries (19). The difference between NBER and BER is that all of the operations implemented in the emergency department would be carried out in the ambulance; therefore, the median of DNT is shortened by 9 min compared with the NBER group. However, not all stroke patients are suitable for this process, for example, those with difficulty to establish venous access, high blood pressure, high blood glucose, and severe trachea management. In this case, it is still necessary to treat them in the emergency room before a head imaging examination. In addition, the situation in which multiple stroke patients arrive at the hospital simultaneously is also not suitable for bypassing emergency mode due to the limited number of thrombolytic physicians on duty. A summary of factors that are not suitable for bypassing emergency mode is presented in Figure 6. However, under the condition of good medical resources and stable vital signs, it is recommended for stroke patients to implement bypassing emergency mode.

After more than 2 years of practice, it can be seen that the Xiangtan model has some differences compared with the Helsinki model and the Melbourne model. The Helsinki model has systematically implemented 12 measures over the years to reduce all possible intravenous thrombolysis delays, while the Melbourne model has learned from the Helsinki model. In our Xiangtan model, we also learned from the strategies of the Helsinki model that can be implemented in developing countries, such as ESM intervention, hospital prenotification, alarm and pre-order of tests, no-delay CT interpretation, delivery of rt-PA on CT table, CT priority and CT transfer, and reduced imaging. However, China is a developing country with the largest population in the world, and many medical policies and medical service systems are still relatively imperfect. The electronic medical records of patients in most medical institutions cannot be obtained in advance like the Helsinki model. In addition, our hospital cannot establish the CT room directly in the emergency room. Besides, we cannot pre-mix rt-PA, because the doctor–patient relationship in China is tense and rt-PA is expensive. If the patients and their families refuse IVT, no one pays for the premixed drug, which will aggravate medical disputes. Therefore, we have conducted some new measures combining our national medical policy and practical experience. We added some new measures, such as the whole recording, and patients are accompanied by stroke physicians and nurses throughout the whole process. Also, payment is made after treatment, there is a standardized communication language, an educational stroke video was made, there is a 24-h work day, stroke and its treatment have been advertised, there is an incentive policy, etc. These measures in our model do not require much economic expenditure to implement. Therefore, developing countries with a large population and difficult and expensive medical treatment can adopt the above measures to reduce intravenous thrombolysis delay in AIS patients.

In conclusion, our study showed that it is possible to limit time to <30 min in DNT for AIS patients by the optimization of thrombolytic strategies suitable for our national conditions. The key to maintaining or even breaking through this goal is that the thrombolytic team works closely together to continuously analyze, evaluate, and improve the factors of delay in the hospital. In addition, the experience with the implementation of reducing in-hospital treatment delays with AIS patients at Xiangtan might provide a reference for more developing countries and regions with similar environments in health care systems.

## Data availability statement

The original contributions presented in the study are included in the article/Supplementary material, further inquiries can be directed to the corresponding authors.

## Ethics statement

The studies involving human participants were reviewed and approved by Medical Ethics Committee of Xiangtan Central Hospital. Written informed consent for participation was not required for this study in accordance with the national legislation and the institutional requirements.

## Author contributions

GY, HX, JZ, and LY designed the study. JX, CL, LL, and FH collected the patient information and data. GY, HX, CL, and JZ analyzed the results and drafted the figure. GY, LY, JX, and LL drafted the tables and manuscript. JZ and LY reviewed and edited. All authors bear responsibility for the integrity and accuracy of the data in the study, contributed to the article, and approved the final manuscript.

## Funding

This study was supported by the Key R&D Plan of Hunan Province (2020SK2078) and the Scientific Bureau of Xiangtan City of Hunan Province of China (grant number SF-YB20201023).

## Conflict of interest

The authors declare that the research was conducted in the absence of any commercial or financial relationships that could be construed as a potential conflict of interest.

## Publisher's note

All claims expressed in this article are solely those of the authors and do not necessarily represent those of their affiliated organizations, or those of the publisher, the editors and the reviewers. Any product that may be evaluated in this article, or claim that may be made by its manufacturer, is not guaranteed or endorsed by the publisher.

## References

[1] Feigin VL Krishnamurthi RV Parmar P Norrving B Mensah GA Bennett DA. Update on the global burden of ischemic and hemorrhagic stroke in 1990–2013: the GBD 2013 study. Neuroepidemiology. (2015) 45:161–76. 10.1159/000441085 26505981 PMC4633282

[2] Li J Liu J Ma Y Peng P He X Guo W. Imbalanced regional development of acute ischemic stroke care in emergency departments in China. Emerg Med Int. (2019) 2019:3747910. 10.1155/2019/3747910 31467718 PMC6701302

[3] Wang W Jiang B Sun H Ru X Sun D Wang L. Prevalence, incidence, and mortality of stroke in China results from a nationwide population-based survey of 480687 adults. Circulation. (2017) 135:759–71. 10.1161/CIRCULATIONAHA.116.025250 28052979

[4] Wardlaw JM Murray V Berge E Del ZG Sandercock P Lindley RL. Recombinant tissue plasminogen activator for acute ischaemic stroke: an updated systematic review and meta-analysis. Lancet. (2012) 379:2364–72. 10.1016/S0140-6736(12)60738-7 22632907 PMC3386494

[5] Sandercock P Wardlaw JM Lindley RI Dennis M Cohen G Murray G. The benefits and harms of intravenous thrombolysis with recombinant tissue plasminogen activator within 6h of acute ischaemic stroke (the third international stroke trial [IST-3]): a randomised controlled trial. Lancet. (2012) 379:2352–63. 10.1016/S0140-6736(12)60768-5 22632908 PMC3386495

[6] Powers WJ Rabinstein AA Ackerson T Adeoye OM Bambakidis NC Becker K. 2018 guidelines for the early management of patients with acute ischemic stroke: a guideline for healthcare professionals from the American heart association/American stroke association. Stroke. (2018) 49:e46–e110. 10.1161/STR.0000000000000158 29367334

[7] Emberson J Lees KR Lyden P Blackwell L Albers G Bluhmki E. Effect of treatment delay, age, and stroke severity on the effects of intravenous thrombolysis with alteplase for acute ischaemic stroke: a meta-analysis of individual patient data from randomised trials. Lancet. (2014) 384:1929–35. 10.1016/S0140-6736(14)60584-5 25106063 PMC4441266

[8] Lindsberg PJ Happola O Kallela M Valanne L Kuisma M Kaste M. Door to thrombolysis: ER reorganization and reduced delays to acute stroke treatment. Neurology. (2006) 67:334–6. 10.1212/01.wnl.0000224759.44743.7d 16864834

[9] Liu Z Zhao Y Liu D Guo ZN Jin H Sun X. Effects of nursing quality improvement on thrombolytic therapy for acute ischemic stroke. Front Neurol. (2018) 9:1025. 10.3389/fneur.2018.01025 30555408 PMC6281878

[10] Greenberg K Maxwell CR Moore KD D'Ambrosio M Liebman K Veznedaroglu E. Improved door-to-needle times and neurologic outcomes when IV tissue plasminogen activator is administered by emergency physicians with advanced neuroscience training. Am J Emerg Med. (2015) 33:234–7. 10.1016/j.ajem.2014.11.025 25498530

[11] Van Schaik SM Van der Veen B Van den Berg-Vos RM Weinstein HC Bosboom W. Achieving a door-to-needle time of 25 min in thrombolysis for acute ischemic stroke: a quality improvement project. J Stroke Cerebrovasc Dis. (2014) 23:2900–6. 10.1016/j.jstrokecerebrovasdis.2014.07.025 25263647

[12] Fonarow GC Zhao X Smith EE Saver JL Reeves MJ Bhatt DL. Door-to-needle times for tissue plasminogen activator administration and clinical outcomes in acute ischemic stroke before and after a quality improvement initiative. JAMA. (2014) 311:1632–40. 10.1001/jama.2014.3203 24756513

[13] Fonarow GC Smith EE Saver JL Reeves MJ Bhatt DL Grau-Sepulveda MV. Timeliness of tissue-type plasminogen activator therapy in acute ischemic stroke: patient characteristics, hospital factors, and outcomes associated with door-to-needle times within 60 min. Circulation. (2011) 123:750–8. 10.1161/CIRCULATIONAHA.110.974675 21311083

[14] Meretoja A Strbian D Mustanoja S Tatlisumak T Lindsberg PJ Kaste M. Reducing in-hospital delay to 20 min in stroke thrombolysis. Neurology. (2012) 79:306–13. 10.1212/WNL.0b013e31825d6011 22622858

[15] McVerry F Hunter A Dynan K Matthews M McCormick M Wiggam I. Country-wide analysis of systemic factors associated with acute ischemic stroke door to needle time. Front Neurol. (2019) 10:676. 10.3389/fneur.2019.00676 31297081 PMC6606974

[16] Huang Q Zhang JZ Xu WD Wu J. Generalization of the right acute stroke promotive strategies in reducing delays of intravenous thrombolysis for acute ischemic stroke: a meta-analysis. Medicine. (2018) 97:e11205. 10.1097/MD.0000000000011205 29924046 PMC6024468

[17] Gu HQ Rao ZZ Yang X Wang CJ Zhao XQ Wang YL. Use of emergency medical services and timely treatment among ischemic stroke. Stroke. (2019) 50:1013–6. 10.1161/STROKEAHA.118.024232 30841820

[18] Puri I Bhatia R Vibha D Singh MB Padma MV Aggarwal P. Stroke-related education to emergency department staff: an acute stroke care quality improvement initiative. Neurol India. (2019) 67:129–33. 10.4103/0028-3886.253636 30860110

[19] Zhang S Zhang J Zhang M Zhong G Chen Z Lin L. Prehospital notification procedure improves stroke outcome by shortening onset to needle time in Chinese urban area. Aging Dis. (2018) 9:426–34. 10.14336/AD.2017.0601 29896430 PMC5988597

[20] Jung S Rosini JM Nomura JT Caplan RJ Raser-Schramm J. Even faster door-to-alteplase times and associated outcomes in acute ischemic stroke. J Stroke Cerebrovasc Dis. (2019) 28:104329. 10.1016/j.jstrokecerebrovasdis.2019.104329 31607439

[21] Lee RS Ok YC Lim JS Lim BC Cho KY Lee MC. Outcome evaluation of intravenous infusion of urokinase for acute ischemic stroke. Chonnam Med J. (2012) 48:52–6. 10.4068/cmj.2012.48.1.52 22570816 PMC3341438

[22] Nguyen-Huynh MN Klingman JG Avins AL Rao VA Eaton A Bhopale S. Novel telestroke program improves thrombolysis for acute stroke across 21 hospitals of an integrated healthcare system. Stroke. (2018) 49:133–9. 10.1161/STROKEAHA.117.018413 29247142 PMC5753819

[23] Lees KR Bluhmki E von Kummer R Brott TG Toni D Grotta JC. Time to treatment with intravenous alteplase and outcome in stroke: an updated pooled analysis of ECASS, ATLANTIS, NINDS, and EPITHET trials. Lancet. (2010) 375:1695–703. 10.1016/S0140-6736(10)60491-6 20472172

[24] Goyal M Almekhlafi M Dippel DW Campbell B Muir K Demchuk AM. Rapid alteplase administration improves functional outcomes in patients with stroke due to large vessel occlusions: meta-analysis of the non-interventional arm from the HERMES collaboration. Stroke. (2019) 50:645–51. 10.1161/STROKEAHA.118.021840 30760169

[25] Gomez CR Malkoff MD Sauer CM Tulyapronchote R Burch CM Banet GA. Code stroke. An attempt to shorten inhospital therapeutic delays. Stroke. (1994) 25:1920–3. 8091434 10.1161/01.str.25.10.1920

[26] Xian Y Smith EE Zhao X Peterson ED Olson DM Hernandez AF. Strategies used by hospitals to improve speed of tissue-type plasminogen activator treatment in acute ischemic stroke. Stroke. (2014) 45:1387–95. 10.1161/STROKEAHA.113.003898 24713527

[27] Demaerschalk BM Kleindorfer DO Adeoye OM Demchuk AM Fugate JE Grotta JC. Scientific rationale for the inclusion and exclusion criteria for intravenous alteplase in acute ischemic stroke a statement for healthcare professionals from the American heart association/American stroke association. Stroke. (2016) 47:581–641. 10.1161/STR.0000000000000086 26696642

[28] Nam HS Han SW Ahn SH Lee JY Choi HY Park IC. Improved time intervals by implementation of computerized physician order entry-based stroke team approach. Cerebrovasc Dis. (2007) 23:289–93. 10.1159/000098329 17199086

[29] Meretoja A Weir L Ugalde M Yassi N Yan B Hand P. Helsinki model cut stroke thrombolysis delays to 25 min in melbourne in only 4 months. Neurology. (2013) 81:1071–6. 10.1212/WNL.0b013e3182a4a4d2 23946303

[30] Zhou Y Xu Z Liao J Feng F Men L Xu L. New standardized nursing cooperation workflow to reduce stroke thrombolysis delays in patients with acute ischemic stroke. Neuropsychiatric Dis Treatment. (2017) 13:1215–20. 10.2147/NDT.S128740 28533683 PMC5431707

[31] Fonarow GC Smith EE Saver JL Reeves MJ Hernandez AF Peterson ED. Improving door-to-needle times in acute ischemic stroke: the design and rationale for the American heart association/American stroke association's target: stroke initiative. Stroke. (2011) 42:2983–9. 10.1161/STROKEAHA.111.621342 21885841

