# Supplementary material for: Reducing intravenous thrombolysis delay in acute ischemic stroke through a quality improvement program in the emergency department
Source: Front Neurol. 2022 Sep 26;13:931193. doi: 10.3389/fneur.2022.931193 (PMC9548581; doi:10.3389/fneur.2022.931193)
Supplement: Supplementary file 1 [file Table_1.DOCX]

**Supporting information**

**Fig. S1. The number of patients with intravenous thrombolysis in the** **Post-intervention group for each month**

**Table S1. Standardized language for communicating with an AIS patient.**

| Four standardized questions for patients that need to be asked before intravenous thrombolysis |
| --- |
| What kind of disease have you had in the past? (Digestive tract, intracranial tumor, renal insufficiency, endocarditis, hemopoietic system)  Have you been taking any medicine? (warfarin, heparin)  Have you had a stroke before?  Have you ever had any trauma or had any surgery before? |
